# Effects of low-sodium bread on dietary compliance and fecal cultivable bacteria in a randomized controlled pilot trial in hypertensive subjects

**DOI:** 10.1186/s40795-024-00838-w

**Published:** 2024-02-21

**Authors:** Carmela Cosola, Francesco Pesce, Maria De Angelis, Valentina Maranzano, Annapaola Zito, Eustacchio Montemurno, Giuseppe Dalfino, Saverio Loiudice, Vincenzo Creanza, Giovanni Pompa, Marco Matteo Ciccone, Giuseppe Grandaliano, Giovanni Stallone, Loreto Gesualdo

**Affiliations:** 1 Dipartimento Di Medicina Di Precisione E Rigenerativa E Area Jonica - (DiMePRe-J), Università Degli Studi Di Bari Aldo Moro, 70124 Bari, Italy; 2 Division of Renal Medicine, Ospedale Isola Tiberina – Gemelli Isola, Roma, Italy; 3 Dipartimento Di Scienze del Suolo, Della Pianta E Degli Alimenti (Di.S.S.P.A.), Università Degli Studi Di Bari Aldo Moro, 70126 Bari, Italy; 4 Cardiovascular Disease Section, Interdisciplinary Department of Medicine, University of Bari Aldo Moro, Bari, Italy; 5 Istituto di Gastroenterologia IRCCS “Saverio de Bellis”, 70013 Castellana Grotte, Italy; 6 Studio Medico Dott. Loiudice, 70022 Altamura, Italy; 7 CPT Europa, Distretto Unico ASL Bari, 70132 Bari, Italy; 8 Dipartimento Di Scienze Mediche E Chirurgiche, Università Degli Studi Di Foggia, 71122 Foggia, Italy; 9 Dipartimento Di Scienze Mediche E Chirurgiche, Policlinico Universitario Agostino Gemelli, 00168 Rome, Italy

**Keywords:** Hypertension, Low-salt diet, Low-sodium bread, Blood pressure, Fecal cultivable bacteria, Randomized controlled pilot trial

## Abstract

High salt intake and compliance to low-sodium (LS) diets are critical in hypertension. Salt reduction in processed foods can help to achieve the target sodium intake. To verify the hypothesis that an innovative LS formulation of a traditional bread could result in a reduction of sodium intake and blood pressure, we performed a 6-month randomized controlled pilot trial on hypertensive patients. We additionally explored the effects of sodium restriction on blood pressure and fecal cultivable bacteria.

Fifty-seven patients were randomized in three groups. Group A (*n* = 19) followed a free diet using standard bread (750 mg Na/100 g), group B (*n* = 18) followed a LS diet (2300 mg Na/die) using standard bread, group C (*n* = 20) followed a LS diet (2300 mg Na/die) using LS bread (280 mg Na/100 g). We measured 24-h urinary sodium, blood pressure, routine parameters, fecal microbial counts (26 patients).

After 6 months, as compared to group A, group C showed a reduction of 24-h urinary sodium excretion (-908 mg/24 h), diastolic pressure (-9 mmHg) and microbial counts of *Bacteroides*, *Porphyromonas*, *Prevotella, Enterobacteriaceae, Staphylococcus, Micrococcus.* These results suggest that LS bread could increase the adherence to a LS diet, reducing sodium excretion, diastolic pressure and abundance of some fecal cultivable bacteria.

**Trial registration **Registration nr. NCT03127553, on 25/04/2017.

**Supplementary Information:**

The online version contains supplementary material available at 10.1186/s40795-024-00838-w.

## Background

High salt intake in developed countries causes raised blood pressure (BP) [1], constituting one main risk factor for cardiovascular disease (CVD) and chronic kidney disease (CKD) [1, 2]. CVD represents the first cause of mortality worldwide [3]. The dose-dependent relationship between salt intake and BP levels [1, 4], and between elevated salt intake and CVD are well acknowledged [5, 6]. For these reasons, reducing salt intake represents a lifestyle change priority to reduce CVD occurrence [1]. Currently, daily salt intake in Western countries ranges around twice the World Health Organization (WHO) recommendation of 5 g/day [1, 7], implying the necessity of a massive worldwide action towards salt reduction, with expected positive outcomes on public health and cost saving [5]. Many efforts have been made and are ongoing, such as in UK, Finland and Canada, where public–private actions to reduce salt intake have been successfully carried out [5].

It is appropriate to report that, while a sodium restriction even beyond the 5 g/day advised by the WHO [7] should be strongly recommended in hypertensive patients, the opportunity of extending the recommendation of a strict low-sodium diet (i.e. around 2.5 g/day) to the general population, as a strategy to prevent hypertension is debated [4, 8, 9], as well as the optimal sodium target for hypertensive patients in order to reduce BP and the risk for cardiovascular events [4, 9, 10].

Nonetheless, there is general agreement on: (i) lowering sodium intake in hypertensive patients, in association with Mediterranean-style regimens such as the DASH diet, is necessary in order to exploit the combined hypotensive effects of potassium and magnesium contained in plant matrices [4, 11]; (ii) reducing salt intake, especially in the long term, requires a great effort by patients and is not always efficiently achieved [5], even after intensive dietary counselling [10], due to low compliance to low-sodium (LS) diets and higher proportion of hidden salt in processed food than in table salt added to preparations, especially in high-income countries [1, 5, 10, 12]. Differently, in low-income countries, discretionary salt contributes for more than 50% to the total salt intake [12]. In developed countries, food industry has a great responsibility in this context. In Italy, bread is a daily-consumed food and contributes, especially in the Southern regions, to the amount of hidden salt in the diet, making it a good target for salt reduction actions.

Salt intake is also related to BP-independent effects. The most well-known are endothelial dysfunction and remodelling [13, 14], atherosclerosis [6], inflammation [6], water retention, increased left ventricular mass, increased risk of stroke and stomach cancer, deterioration of renal function, renal stones and osteoporosis, asthma and obesity [5]. Recently, alterations of gut microbiota composition and diversity following elevated salt intake have been reported [15, 16], with reduction of probiotic *Lactobacillus*spp [17].. Moreover, accumulating evidence indicates a role of microbiota in BP regulation and hypertension aetiology [18, 19]. Anyway, there are no current available studies exploring the effects of dietary sodium restriction on gut microbiota composition. PROALIFUN was an Italian Ministry of University and Research-funded project, aimed at designing and clinically validating innovative protocols for the production of functional foods, in collaboration with food industry and public research organisms. The present study is part of this project, carried on with a private–public collaboration approach, already successful in demonstrating the healthy properties of other functional foods [20, 21]. We designed this trial to verify if an innovative formulation of a traditional South Italy PDO (Protected Designation of Origin) bread (“Pane di Altamura” by Oropan SPA, Italy) containing 50% less sodium than the conventional one (in accordance to the Italian National Programme “Guadagnare Salute”) could improve the adherence to a LS diet and be effective in achieving the clinical target of BP reduction, in a group of hypertensive subjects. Secondly, we explored some BP-independent effects of sodium restriction: cultivable bacterial composition and endothelial, metabolic and renal function parameters.

## Methods

### Patients and study design

We carried out a multicenter, open-label randomized controlled pilot trial, according to Helsinki Declaration (IV Adaptation) and European Guidelines for Good Clinical Practice. The trial was designed in order to adhere to CONSORT guidelines. Being a pilot study, we established a minimum target of 12 participants for each group [22]. The study protocol was approved by the Ethical Committee of the Azienda Ospedaliero-Universitaria Consorziale Policlinico of Bari, Italy (Authorization nr. 1644/2014, 16st December 2014) and registered in the ClinicalTrials.gov registry database (registration nr. NCT03127553, on 25/04/2017). Hypertensive patients were enrolled according to the following inclusion/exclusion criteria: (i) Inclusion: arterial hypertension stage I-II (BP between 130/80 mmHg and 145/95 mmHg), aged between 50 and 80 years, stable control of BP, estimated glomerular filtration rate (eGFR) > 60 ml/min/1.73m [2], signed informed consent; (ii) Exclusion: diuretic therapy, need for more than three antihypertensive drugs, proteinuria > 1 g/day, celiac disease, systemic inflammatory diseases, suspect/clinical diagnosis of malignancy, chronic liver disease, corticosteroids/immunosuppressive therapy, previous acute CVDs (myocardial infarction, stroke), psychiatric conditions reducing the compliance to protocols. Primary outcome: reduction of sodium intake, measured as 24-h urinary sodium concentration. Secondary outcomes: reduction of BP, gut microbiota modulation, evaluation of endothelial stress through flow-mediated dilation (FMD). All the outcomes were evaluated after six months of intervention.

Volunteers were recruited in two different ways. The Bari Unit recruited patients amongst the hypertensive patients assisted by two general practitioners’ (GP) offices (Dr. Loiudice private office—Altamura Italy, and the associated general practitioner clinic “CPT Europa” – Bari Italy). The GPs cooperated to this study by informing the potentially eligible patients and by collecting an informal consent to be contacted by the medical personnel involved in the study for eligibility assessment. The Foggia Unit recruited patients attending the Unit of Hypertension of the Department of Surgical and Medical Sciences. The study was carried out at outpatient centers of Nephrology and Cardiovascular Disease Units, Department of Emergency and Organ Transplantation – University of Bari (*n* = 26 patients enrolled) and of Nephrology Unit, Department of Surgical and Medical Sciences – University of Foggia (*N* = 31 patients enrolled). The recruitment started in December 2014 and ended in June 2015. The study was planned to end before summer, in order to avoid important losses of sodium through sweating. Moreover, to counteract the shift of environmental temperature from December to June, all patients were enrolled in the same month and completed the study in the same month. In this way, seasonal variation of Na excretion are compensated by the parallel arm design of the study.

### Randomization and nutritional counseling

At enrollment, each participant signed a written informed consent. Patients belonging to each participating center were randomly allocated into three study arms (A, B and C) in a 1:1:1 ratio by an independent researcher by using a software, with gender and smoking as blocking factors. In both centers, patients were enrolled by a physician and assigned to nutritional intervention by a dietitian. After a literature review and evaluation of feasibility of LS diet, also considering the salt content in experimental and standard bread [2, 4, 10], a sodium reduction target of 2300 mg/day was set.

Group A represented the control group, with patients undergoing a six-month free-diet (FD), including the use of the standard “Oropan” Altamura bread (containing 750 mg Na for 100 g product). Patients randomized in the group B followed a six-month LS diet, including the use of standard “Oropan” Altamura bread (containing 750 mg Na for 100 g), while patients randomized in the group C followed a six-month LS diet, including the use of the innovative LS “Oropan PANDELCUORE” Altamura bread (containing 280 mg Na for 100 g). Group B and C diets were normocaloric, normoproteic, LS (2300 mgNa/day), Mediterranean-style (60% carbohydrates, 15% proteins, 25% fats), including 5 daily servings of fruits and vegetables, 120 g of experimental (either standard or LS) bread, 1 daily serving of milk/yogurt, 1 daily serving of pasta/whole-grain cereals, 1 daily serving of a protein dish, according to the following weekly frequency: 2–3 times legumes, 1–2 times fish, 1–2 times meat (preferably white), 1 time eggs. Patients in both groups received differential indications about the allowed amount of table salt (respectively, 2.5 g and 4 g) in order to respect the total daily amount of salt prescribed. The nutritional composition of the two breads is reported in Supplementary Table 2. At the enrollment and throughout the study, each patient received, twice a week, a supply of either standard or LS bread, depending on the randomization, providing an amount of three bread slices (120 g) per day.

At the beginning (T0) and at the end (T6) of the study period, each patient underwent medical examination, including: resting office BP measurement, height and weight measurement, 12-h fasting venous blood and 24-h urine collection for determining urinary and blood chemistry profile, stool collection, FMD analysis. Moreover, they underwent a nutritional visit including anthropometric and food intake evaluation (by a food frequency questionnaire and 24 h recall). All the data were registered on an electronic case report form. No incentive was provided to the volunteers, while the bread was provided free of charge.

### Blood and urine chemistry

Fasting morning blood samples were used to measure serum glucose using Siemens enzymatic method (Dimension Vista 1500, Siemens Health Diagnostics, Deerfield, IL), glycated haemoglobin (HbA1c) levels by high-performance liquid chromatography (BioRad D10, Pratteln, Switzerland) and creatinine by enzymatic method (Dimension Vista 1500, Siemens Health Diagnostics, Deerfield, IL). Additional aliquots of serum were obtained, after centrifugation, for each patient/time point and stored at − 80 °C until use. Moreover, 24-h urine samples were collected to measure the sodium concentration. Briefly, timed 24-h urine specimen collection was started in the morning before the visit, after discarding first morning urine sample. The entire volume of urine was collected in the clean, unused container with 2–3 L volume. Total volume of the collection was measured. According with the standard procedure, urinary sodium was measured using ion selecting electrode method by V-Lyte IMT autoanalyser (Dimension Vista® 1500, Siemens Healthcare Global, Erlangen, Germany).

### Blood pressure and Flow-mediated dilation of brachial artery

Office BP was measured in the supine position using a sphygmomanometer with an appropriate cuff on the left arm; the average of two readings was used for statistical analysis. Hypertension was defined as BP > 130/80 mmHg or currently on antihypertensive treatment [23].

In the Bari unit subgroup (*n* = 26 patients), endothelium-dependent vasodilatation FMD of the brachial artery was non-invasively assessed, using high-resolution ultrasound in a quiet, air-conditioned environment (22–24 °C), on the same day of the visit and blood sampling. The patients fasted for at least 8–12 h. The study was performed using an image analysis system software, certified by the CNR of Pisa (MVE II), as previously described [21]. Briefly, subjects were placed in supine position, a sphygmomanometer cuff was inserted distally to the brachial artery and image acquisition was made before, during and after an ischemic stimulus. The FMD was calculated as described elsewhere [24]. The measures of FMD showed good reproducibility, with an ICC of 0.95.

### Enumeration of cultivable bacteria

Fecal samples were collected at each time point by the Bari unit subgroup (*n* = 26 patients, group A *n* = 9, group B *n* = 8, group C *n* = 9). After collection, samples were immediately diluted with Amies Transport medium (Oxoid LTD, Basingstoke, Hampshire, UK) (ca. 15 g, 1:1 wt/wt), under anaerobic conditions (AnaeroGen, Oxoid LTD, Basingstoke, Hampshire, UK). Samples mixed with Amies Transport medium were immediately analysed (plate counts). Fecal samples (5 g) were mixed with 45 ml sterilized physiological solution and homogenized. Counts of viable bacterial cells were carried out as described elsewhere [25]. The following selective media were used: Wilkins-Chalgren anaerobe agar (total anaerobes); Plate count agar (total aerobes and anaerobes); MRS agar (Enterococcus, Lactobacillus and Leuconostoc); Slanetz and Bartley (Enterococcus); Rogosa agar, plus 1.32 ml/l of glacial acetic acid (Lactobacillus); M17 (Lactococcus and Streptococcus); Baird Parker (Staphylococcus); Reinforced Clostridial Medium supplemented with 8 mg/L novobiocin, 8 mg/L colistin (Clostridium); Wilkins-Chalgren anaerobe agar, plus GN selective supplements and sheep blood defibrinated (Bacteroides, Porphyromonas and Prevotella); MacConkey agar No2 (Enterobacteriaceae); Chromocult (total coliform) (Merk, Darmstadt, Germany, Europe); GSP agar (Sigma-Aldrich, St. Louis, MO), plus penicillin-G (60 g/l) (Pseudomonas, Aeromonas); Bifidobacterium agar modified (Bifidobacterium) (Becton Dickinson, Le Pont de Claix, SA, France). Except for Bifidobacterium agar modified, Chromocult and GSP agar, all media were purchased by Oxoid Ltd (Basingstoke, Hampshire, UK).

### Community-level catabolic profiles

Carbon source utilization patterns of the fecal microbiota were assessed using Biolog 96-well Eco micro-plates (Biolog, Inc., Hayward, CA). Micro-plates contained a variety of carbon sources (carbohydrates, carboxylic acids, polymers, amino acids, amines, and miscellaneous substrates), and were assessed in triplicate. Five grams of feces mixed with Amies Transport medium (1:1) were treated as previously described [20]. The microbial suspension was diluted into sterile chloride solution and distributed into each a 96-wells Biolog Eco micro-plates. They were incubated at 30 °C in the dark on slow stirring, and a micro-plate reader (Biolog Microstation) was used to measure 590 nm absorbance every 24 h. Three indices were determined [26]. Shannon’s diversity (H’), substrate richness (S), and substrate evenness (E) were calculated, respectively indicating the substrate utilization pattern, the number of different substrates used and the equitability of activities across all utilized substrates. They were calculated as reported elsewhere [20].

### Statistical analysis

24-h urinary sodium and systolic and diastolic BP were defined in terms of variation (T6-T0) and expressed as medians and interquartile range (IQR). Data distribution was assessed by D'Agostino&Pearson normality test. Normally distributed data are presented as mean ± standard deviation, non-normally distributed data as median and interquartile range. Differences between quantitative variables were analyzed by unpaired Student t-test (normally distributed data) or Mann–Whitney (non-normally distributed data) test as appropriate. Additionally, one-way ANCOVA was performed to compare the effectiveness of three nutritional interventions on sodium excretion, using baseline urinary sodium, systolic and diastolic BP as covariates. Levene’s test and normality checks were carried out and the assumptions met. *p*-values from microbial counts analysis were adjusted for multiple comparisons using the false discovery rate (FDR). *p*-values < 0.05 were considered significant. All the analyses were performed using SPSS software (version 21; IBM, Armonk, NY).

## Results

### Baseline characteristics of the enrolled population

The recruitment started in December 2014 and ended in June 2015. After screening, 57 eligible patients (35 males, 22 females) with stable BP control in pharmacological therapy with ACE inhibitors, calcium channel blockers or angiotensin II receptor blockers, were enrolled in the two participating centers and randomized into the three study arms (19 group A, 18 group B, 20 group C). One patient in group B and two patients in group C abandoned the study during the follow-up for personal reasons (Fig.S1). 54 patients completed the study and their data were included in the final analysis. Baseline clinical characteristics of the study population did not significantly differ between the groups (Table 1).

**Table 1:** Baseline** c**linical characteristics of enrolled patients

	**Group**		
	**A (*n* = 19)**	**B (*n* = 17)**	**C (*n* = 18)**
**Age (y)**	66.6 ± 6.3	63.2 ± 6.9	63.6 ± 6.2
**BMI**	28.4 ± 3.2	28.8 ± 5.1	27.8 ± 3.6
**Systolic BP (mmHg)**	138.4 ± 10.4	134.6 ± 9.8	139.3 ± 10.3
**Diastolic BP (mmHg)**	85.4 ± 6.8	84.1 ± 5.7	88.9 ± 5.7
FMD (%) *	7.4 ± 1.4	5.8 ± 2.0	5.6 ± 2.0
**HbA1c (mmol/mol)**	39.4 ± 6.1	38.5 ± 7.8	41.1 ± 10.6
**Serum creatinine (mg/dl)**	0.9 ± 0.2	0.9 ± 0.2	0.8 ± 0.2
**MDRD (mL/min/1.73 m2)**	77.3 ± 14.9	86.5 ± 21.6	87.1 ± 13.4
**Urinary Sodium (mg/24 h)**	3661 (2620,4071)	3408 (2695,4427)	3793 (3399,4982)

^*^Performed on a subgroup of 26 patients

*Abbreviations*: *BMI* Body Mass Index, *BP* Blood Pressure, *FMD* Flow-Mediated Dilation, *HbA1c* Glycated Hemoglobin, *MDRD* Modification of Diet in Renal Disease

Age, BMI, systolic/diastolic BP, FMD, HbA1c, serum creatinine, MDRD are expressed as mean ± standard deviation, urinary sodium as median and interquartile range

### Reduction of urinary sodium excretion and diastolic blood pressure in group C

**Fig. 1:**
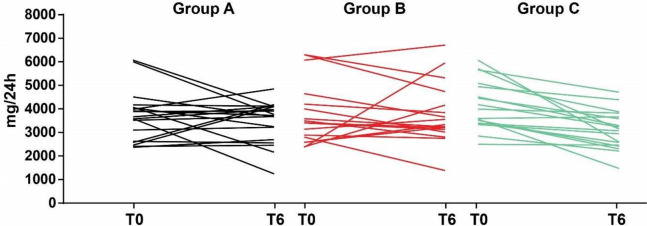
Change of urinary sodium excretion after the nutritional treatments in the three study groups**.** Each line represent individual urinary sodium levels of patients belonging to groups A, B and C before (T0) and after (T6) the nutritional treatments. After six months, a significant reduction of sodium excretion was observed in group C patients [F = 5.259, *p* = 0.027]

After the six-months dietary intervention, a significant reduction of sodium excretion was observed in group C versus group A [-908 (-1219,-370) group C vs 23 (-846,397) group A mg/24 h, *p* = 0.01] and versus group B [-908 (-1219,-370) group C vs -276 (-987,640) group B mg/24 h, *p* = 0.04] (Fig. 1, Table 2).

**Table 2:** Effect of the intervention on urinary sodium excretion and on BP

	**Urinary sodium**			**Systolic BP**			**Diastolic BP**		
	**A**	**B**	**C**	**A**	**B**	**C**	**A**	**B**	**C**
**Values after treatment**	3720	3335	3112	133.8	132.7	130.6	83.2	86.7	81.6
**IQR/St Dev**	(2699,4094)	(3072,4451)	(2450,3703)	14.5	13.2	9.1	10.0	10.1	8.1
**Median of changes**	23	-276	-908	-5	-5	-10	0	5	-9
**IQR**	(-846,397)	(-987,640)	(-1219,-370)	(-12.5,5)	(-10,17)	(-20,0)	(-6.5,2.5)	(-5,10)	(-13.8,-1.3)
**MW vs A**		0.74	0.01		0.79	0.19		0.33	0.04
**MW vs B**			0.04			0.19			0.01

*Abbreviations*: *BP* Blood Pressure, *IQR* Interquartile range, *MW* Mann–Whitney test. Values of urinary sodium (mg/24 h) and changes to baseline of all the parameters (urinary sodium, systolic and diastolic BP) are expressed as median and interquartile range, values of systolic/diastolic BP (mmHg) are expressed as mean ± standard deviation

The LS diet in group B did not result in a significant difference in sodium excretion between groups A and B at the end of the treatment (Table 2). The result was confirmed by ANCOVA analysis, showing a significant difference in urinary sodium excretion [F = 5.259, *p* = 0.027] between the groups.

Consistently with urinary sodium trends, a significant reduction of diastolic BP was evidenced in group C versus group A [-9 (-13.8,-1.3) group C vs 0 (-6.5,2.5) group A mmHg, *p* = 0.04] and versus group B [-9 (-13.8,-1.3) group C vs 5 (-5,10) group B mmHg, *p* = 0.01] (Table 2), although lacking confirmation by ANCOVA analysis. At T6, no significant differences in diastolic BP between groups A and B, nor differences in systolic BP between any group were detected (Table 2).

Endothelial function, nutritional status, glycemic metabolism and renal function remained stable after the dietary intervention in each of the three study arms (Supplementary Table 1).

### Changes in the amount of specific fecal cultivable bacteria and community-level catabolic profiles after the LS dietary counseling

**Fig. 2:**
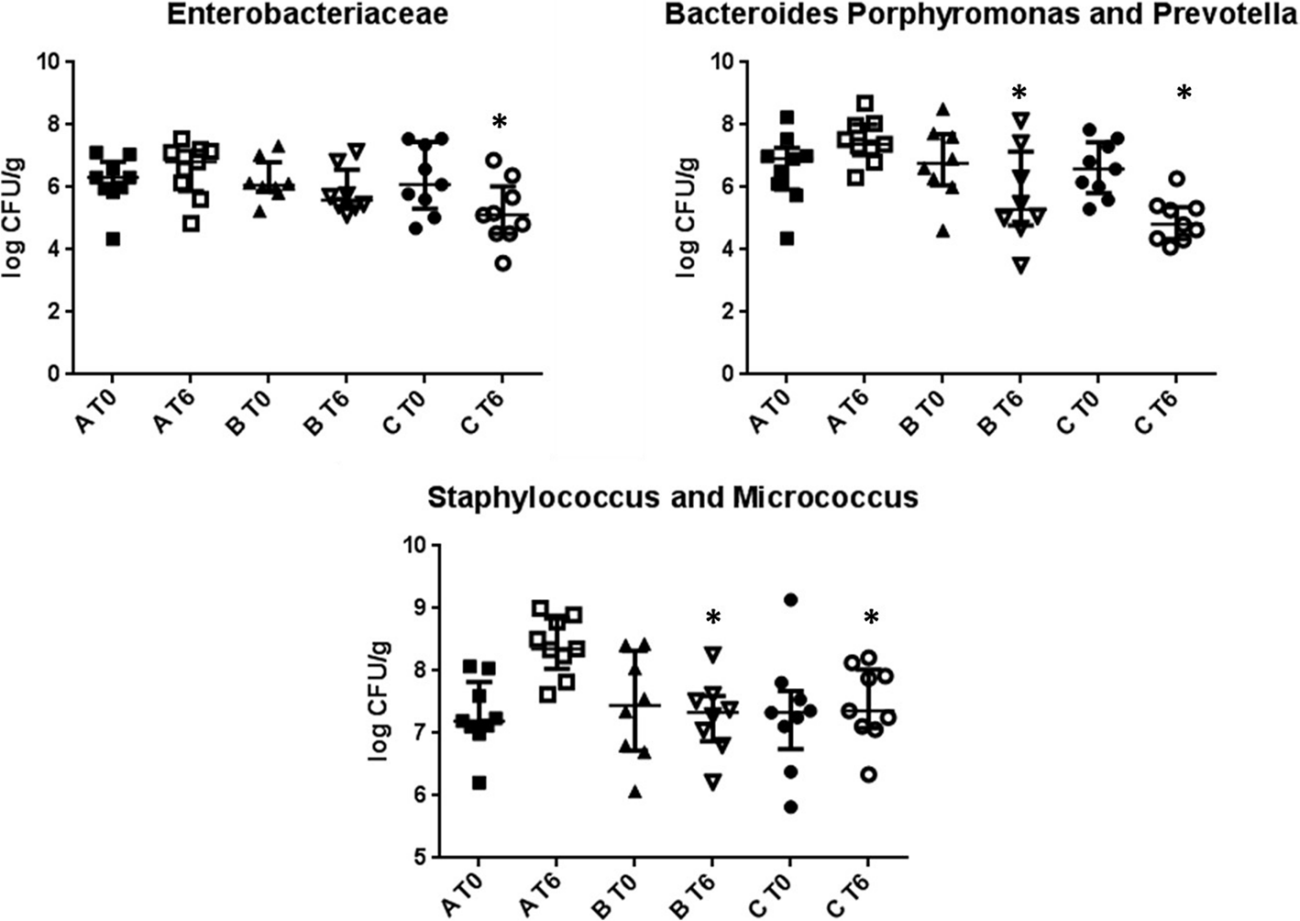
Change of some cultivable bacteria after the nutritional treatments in the three study groups**.** Data are the means of three independent experiments (*n* = 3). Data are represented as median and interquartile range (75th and 25th percentile). *p < 0.05 vs group A

At baseline, the microbial counts of presumptive *Clostridium*, *Enterobacteriaceae*, *Bacteroides*, *Porphyromonas* and *Prevotella*, *Staphylococcus* and *Micrococcus*, *Enterococcus*, *Aeromonas* and *Pseudomonas*, *Lactobacillus*, *Bifidobacterium* did not significantly differ between groups. At T6, *Enterobacteriaceae* in group C were significantly decreased in comparison to group A [group C 5.11 (4.52,6.03) vs group A 6.31 (5.90,6.82) logCFU/g, *p* = 0.02] (Fig. 2). Moreover, microbial counts of *Bacteroides, Porphyromonas* and *Prevotella* in both groups B [5.29 (4.78,7.15) logCFU/g] and C [4.81 (4.34,5.37) logCFU/g] were significantly decreased as compared to the group A [7.38 (7.03,8.01) *p*-values vs group B *p* = 0.02; vs group C *p* < 0.001] (Fig. 2). Similarly, *Staphylococcus* and *Micrococcus* in both groups B [7.33 (6.87,7.69) logCFU/g] and C [7.36 (7.09,8.03) logCFU/g] were significantly decreased at T6 as compared to the group A [8.35 (8.03,8.84) *p*-values vs group B and C *p* = 0.01] (Fig. 2). *Clostridium*, *Enterococcus*, *Lactobacillus*, *Aeromonas* and *Pseudomonas*, *Bifidobacterium* were not affected by nutritional treatments, although *Clostridium* showed a trend towards reduction (Supplementary Fig. 2).

Community-level catabolic profiles were lower (*p* < 0.05) in groups B and C compared to group A at T6. In detail, Shannon’s diversity (H’) indexes at the end of the treatment were 16, 6 and 12 in groups A, B and C, respectively. The substrate richness (S) was the lowest in B group (1.48 group B vs 2.76 group A, *p* = 0.01). No statistically significant difference was found between S values of A and C groups at T6.

## Discussion

In this six-month randomized controlled pilot study, carried out in hypertensive patients, we showed that a LS bread used in the context of a LS diet reduced urinary sodium excretion and diastolic BP, as compared to the FD and the LS diet including a standard bread. The latter did not significantly reduce sodium intake and BP, suggesting that the functional bread together with the LS diet are effective in reducing sodium intake and BP. Moreover, some cultivable fecal bacteria were differentially modulated in patients following LS diets, compared to FD.

It is acknowledged that BP can be modulated by several micronutrients such as sodium, potassium, magnesium, nitrate, being this effect more evident when micronutrients are provided by food matrices rather than supplements [11, 27]. Particularly, the beneficial effects of sodium restriction in hypertension are well established [1, 4, 10, 12]. Sodium intake is dramatically high in Western countries, being the massive presence of industrial food in Western diets responsible for this: only 25% of daily sodium intake comes from discretionary salt in developed countries [12], implying that sodium restriction is mainly out of people’s control. Indeed, efforts to control salt intake are often damped by poor adherence to LS diets [1, 10], mainly due to low palatability and scarce market availability of LS foods. Many international programs of salt reduction have been successfully experimented in the past [5], while European Union launched a salt reduction initiative [28]. Italian Ministry of Health adopted these recommendations by launching the National Program “Guadagnare Salute”, to which the functional bread tested in this trial belongs [29].

Traditional Southern Italy bread, such as the PDO “Pane di Altamura”, contains a higher quantity of sodium if compared with other Italian breads [30], i.e. 750 mg of sodium for 100 g of product. The functional LS Altamura bread contains about 50% less sodium with comparable palatability, as assessed by internal panel studies and informal reporting by participants of the trial.

Some studies included the use of LS functional foods [10, 31–34] or potassium-based salt [35–37] or supplements [38–40] to achieve the clinical goals linked to salt restriction. To our knowledge, other studies carried on with a LS functional food compared to the conventional one, in the context of a defined LS diet, designed in order to demonstrate a differential effectiveness in facilitating the adherence to the latter, are not available. One interesting study similar to the present one included 89 Danish families, randomized into three arms (A—sodium-reduced bread, B—sodium-reduced bread and dietary counseling, C—regular sodium bread), and showed the effectiveness of the combination of the LS bread and dietary counselling in reducing sodium intake. Differently to our study though, it did not explore clinical parameters such BP, and it did not separately compare the effectiveness of the dietary counselling alone or in combination with the LS bread [41]. The design of this pilot study allowed drawing two initial conclusions, as already reported in an oral communication at 54th ERA-EDTA Congress [42]. Firstly, patients of LS group B failed to achieve both sodium excretion and BP reduction goals. The clinical results were not distinguishable from the control group A, although a trend towards reduction of urinary sodium excretion was observed. The second, most important observation, concerns the difference both in sodium excretion and in diastolic but not systolic BP levels between the two LS intervention arms B and C. Patients belonging to these groups, indeed, followed six-month LS diets, both theoretically providing an overall daily intake of 2300 mg Na. Results were unequal in practice, since urinary sodium levels and diastolic BP showed a significant decrease only in group C. Taken together, such data suggest a better compliance to the dietary sodium restriction by the latter.

We also explored possible effects of dietary sodium restriction on some fecal cultivable bacteria. The intimate connection between many pathological states, including hypertension, gut microbiota and extra-intestinal organ function is progressively emerging [18, 43, 44]. Indeed, dysbiosis has been reported in correlation with hypertension by some studies [45, 46]. In animal hypertension models, changes in the Firmicutes and Bacteroides ratio have been reported [45, 47]. As for many human physiological mechanisms, gut microbiota seems to impact BP regulation, salt sensitivity and hypertension through the release of active metabolites [18, 19]. In confirmation of this, some experimental studies on gut microbiota modulation through antibiotics, probiotics, prebiotics and fecal transplantation reported an effect on BP regulation [18, 45–48]. In particular, antibiotic treatments affecting the gut microbiota, especially the Firmicutes and Bacteroidetes components, reduced the high BP in spontaneously or induced hypertensive rats [45]. Beyond functional foods and supplements [20, 21, 43], diet is an important modulator of gut microbiota composition and metabolism and this evidence is progressively encouraging the adoption of functional nutritional approaches in the management of many chronic pathologies [18, 43–45]. In this regard, only a few studies investigated the effects of elevated dietary salt intake on intestinal microbiota. Two recent animal studies reported negative effects of high-salt diets on gut microbiota composition [15] and diversity [16] and on protein digestion [15], collectively increasing Firmicutes/Bacteroidetes ratio, decreasing *Lactobacillus* abundance and reducing alpha and beta diversity indexes. Interestingly, a pilot study with a 14-days high salt challenge in humans evidenced a reduced intestinal survival of probiotic *Lactobacillus* spp. and increased both systolic and diastolic BP, supporting a role of microbiota in BP regulation [17].

Anyway, as far as we know, the present study is the first reporting the effects of reduction of dietary sodium intake on bacterial abundance, in a human trial on hypertensive subjects. Another study showed an increase in circulating short-chain fatty acids following a LS diet, failing to report the effects on gut microbial composition [49]. Our data show that sodium restriction drives a decrease of cultivable *Bacteroides*, *Porphyromonas* and *Prevotella, Enterobacteriaceae, Staphylococcus* and *Micrococcus*. The lowering of the amount of fecal bacteria by the LS diet was also confirmed by the lower community-level catabolic profiles associated to B and C groups. However, Shannon’s diversity was restored after six months in C group, indicating a new balance of bacterial metabolic pathways. Interestingly, the LS diet did not affect the relative amount of some health-promoting bacteria (such as *Lactobacilli* and *Bifidobacteria*), previously shown capable to produce anti-hypertensive bioactive peptides [48], both in animals and in humans [47].

Our study has several limitations: firstly, it is a pilot trial with a limited sample size. This can explain the lack of a clear effect on BP, in parallel to the significant reduction of sodium excretion. Only diastolic pressure shows a trend towards reduction in group C in comparison to the control group A, although this result becomes not significant after the ANCOVA analysis. A larger sample size could probably have allowed observing a significant clinical effect. Secondly, in this study we applied a cultural-dependent approach, evidencing a modulation of some cultivable bacterial genera. Certainly, a metagenomic approach could have allowed us to gain a comprehensive overview of gut microbiota modulation following dietary sodium restriction. Third, we used FFQ and 24-h recall to check adherence to the prescribed dietary protocols, verifying that the compliance to diets was good, provided that what stated by the patients corresponded to the truth. This is, in general, a limitation in using instruments based on self-reported data. This self-reported good compliance to diets allowed us to conclude that the differential results obtained in the 24 h urine Na excretion and in blood pressure could be reasonably attributed to: 1. the different breads, and 2. to the discretionary salt added to the preparation of food. Indeed, the latter is a factor that heavily affects the palatability of food, and it is difficult to verify by the 24 h-recall, although patients in group B declared adherence to prescription of 2.5 g of salt daily. The impossibility of demonstrate this aspect with certainty is surely an important limitation of the study. We also evaluated if the shift towards a strict Mediterranean Diet profile would also affect the reduction of BP, but we excluded this possibility. Indeed, we analyzed the FFQs of the participants, by calculating the 14-items Mediterranean diet Adherence Score [50], and no significant difference among the three groups was found. This observation further confirmed that the observed reduction of BP was due to the decreased dietary salt (provided by the bread and LS diet in the Mediterranean style). At any rate, the present study appears to be the first in literature exploring the differential adherence to a dietary salt restriction by comparing the use of a LS functional food vs a conventional one, in the context of a LS diet. Moreover, the present study reports for the first time a modulation of some fecal cultivable bacteria following dietary sodium restriction, although the biological and physiological significance for this relationship needs to be further investigated.

## Conclusions

In conclusion, despite the limited sample size of our pilot trial, our results suggest that the introduction of a LS functional bread in the context of a LS diet is helpful in improving the adherence to dietary sodium restriction, in achieving sodium intake and possibly BP reduction. Our results show additional effects of sodium restriction at intestinal level, with a modulation of specific fecal cultivable bacteria.

Our public–private collaboration, set in a road already tracked by pioneering examples carried on in UK and Finland [5], focused on an Italian traditional, high-sodium daily used food, such as bread. The present results support the extension on a larger trial, with the potential of being transposed in different geographical contexts. Future confirmation of these results could provide additional evidence to encourage food authorities and industry to design innovative formulations of traditional and industrial high-salt foods.

### Supplementary Information

**Additional file 1: Figure S1. **Study flow diagram. CONSORT® 2010 flow diagram for randomized studies, representing the total number of people assessed for eligibility, enrolled, allocated into the three study arms after randomization and analysed. **Figure S2. **Fecal cultivable bacteria not affected by LS diet. Swarm plots of cultivable genera (log CFU/g) not affected by the nutritional treatments (*Clostridium*, *Enterococcus, Aeromonas *and* Pseudomonas, Lactobacillus, Bifidobacterium*) are shown. Data are the means of three independent experiments (n = 3). Data are represented as median and interquartile range (75th and 25th percentile).

**Additional file 2: Supplementary Table 1. **Effect of the intervention on clinical and anthropometrical parameters.**Supplementary Table 2.** Nutritional composition of the breads used in the study (values referred to 100g of product).

## Data Availability

The datasets generated during and/or analysed during the current study are available from the corresponding author on reasonable request.

## Abbreviations

LS: Low-sodium
BP: Blood pressure
CVD: Cardiovascular disease
CKD: Chronic kidney disease
WHO: World Health Organization
PDO: Protected Designation of Origin
eGFR: Estimated glomerular filtration rate
GP: General practitioners
FMD: Flow-mediated dilation
FD: Free-diet
HbA1c: Glycated haemoglobin
IQR: Interquartile range
FDR: False discovery rate
BMI: Body mass index
MDRD: Modification of diet in renal disease
