# Supplementary material for: Effects of low-sodium bread on dietary compliance and fecal cultivable bacteria in a randomized controlled pilot trial in hypertensive subjects
Source: BMC Nutr. 2024 Feb 21;10:31. doi: 10.1186/s40795-024-00838-w (PMC10882934; doi:10.1186/s40795-024-00838-w)
Supplement: Supplementary file 2 — Additional file 2: Supplementary Table 1. Effect of the intervention on clinical and anthropometrical parameters.Supplementary Table 2. Nutritional composition of the breads used in the study (values referred to 100g of product). [file 40795_2024_838_MOESM2_ESM.doc]

**Supplementary Figure 1**


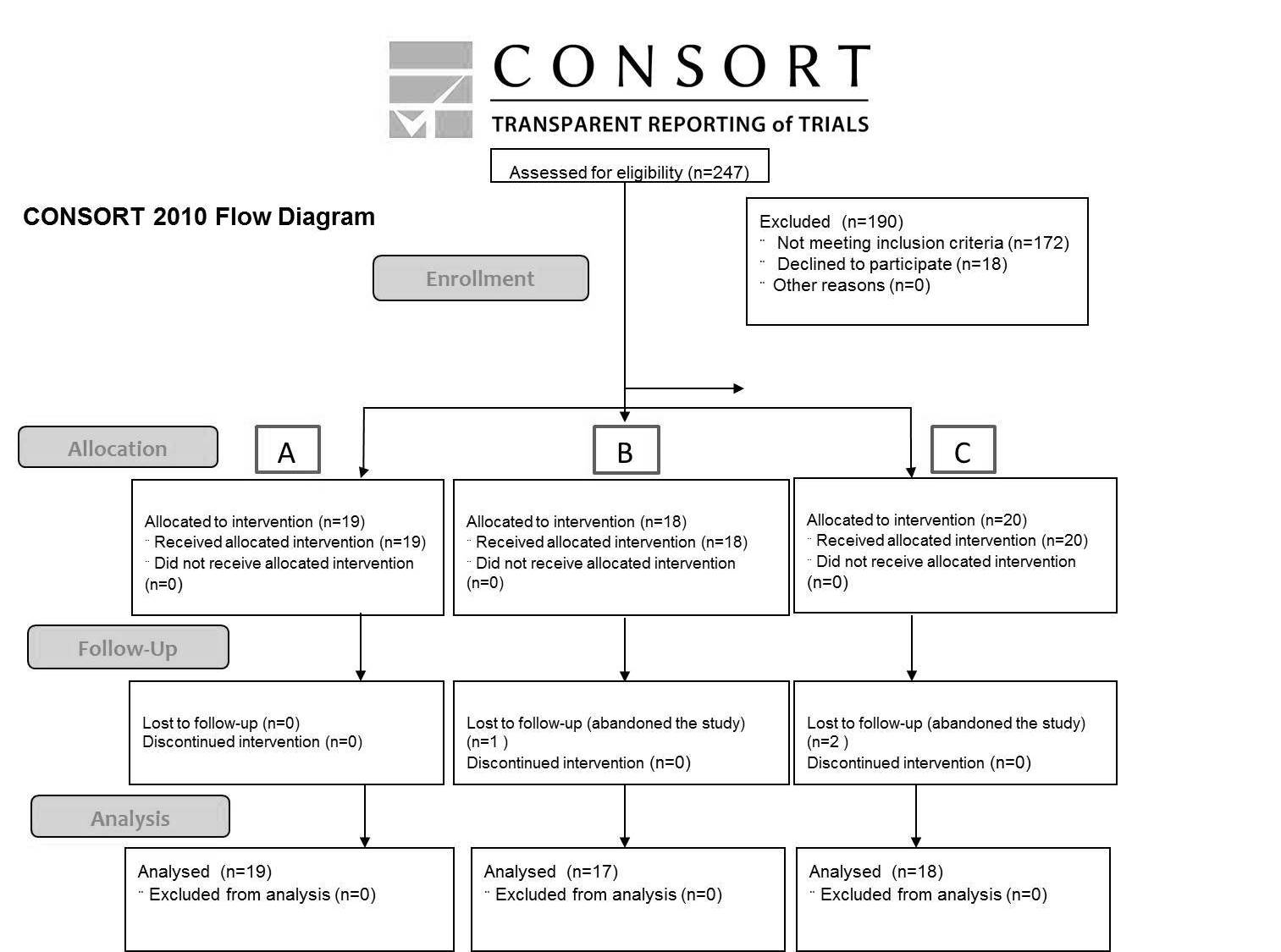


**Figure S1. Study flow diagram.** CONSORT® 2010 flow diagram for randomized studies, representing the total number of people assessed for eligibility, enrolled, allocated into the three study arms after randomization and analysed.

**Supplementary Figure 2**

**
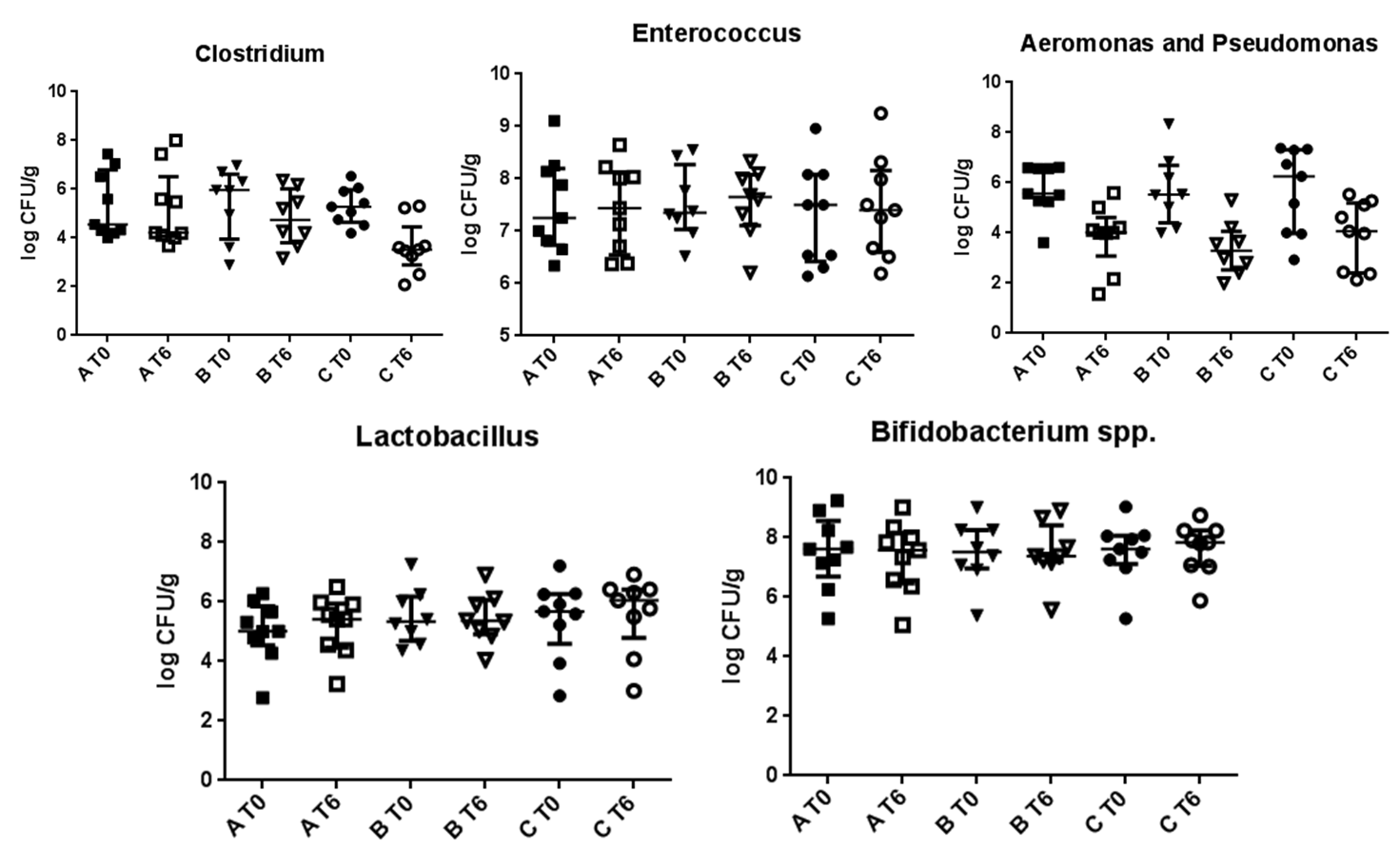
**

**Figure S2. Fecal cultivable bacteria not affected by LS diet.** Swarm plots of cultivable genera (log CFU/g) not affected by the nutritional treatments (*Clostridium*, *Enterococcus, Aeromonas* and *Pseudomonas, Lactobacillus, Bifidobacterium*) are shown. Data are the means of three independent experiments (n = 3). Data are represented as median and interquartile range (75th and 25th percentile).

**Supplementary Table 1: Effect of the intervention on clinical and anthropometrical parameters**

|  |  | **mean** | **p A vs B** | **p A vs C** | **p B vs C** |
| --- | --- | --- | --- | --- | --- |
| **FMD (%)** | A | 9.7±2.5 | - | - | - |
|  | B | 10.4±2.6 |  |  |  |
|  | C | 9.8±3.9 |  |  |  |
| **BMI** | A | 28.6±3.4 | - | - | - |
|  | B | 28.5±5.2 |  |  |  |
|  | C | 27.3±3.1 |  |  |  |
| **HbA1c (mmol/mol)** | A | 39.9±6.2 | - | - | - |
|  | B | 39.5±3.0 |  |  |  |
|  | C | 38.5±11.0 |  |  |  |
| **Serum creatinine (mg/dl)** | A | 0.9±0.2 | - | - | - |
|  | B | 0.9±0.2 |  |  |  |
|  | C | 0.8±0.2 |  |  |  |
| **MDRD (mL/min/1.73 m^2^)** | A | 79.8±16.4 | - | - | - |
|  | B | 89.6±22.7 |  |  |  |
|  | C | 91.2±22.8 |  |  |  |

Abbreviations: Flow-Mediated Dilation FMD, Body Mass Index BMI, Glycated Hemoglobin HbA1c, Modification of Diet in Renal Disease MDRD. Data are expressed as mean ± standard deviation.

**Supplementary Table 2: Nutritional composition of the breads used in the study (values referred to 100g of product)**

|  | **Normal Bread** | **Low-Sodium Bread** |
| --- | --- | --- |
| **Calories** | 1264 kJ / 298 kcal | 1235 kJ / 291 kcal |
| **Protein** | 11 g | 11 g |
| **Carbohydrate** | 59 g | 59 g |
| **Sugars** | 0 g | 0 g |
| **Total Fat** | 1 g | 1 g |
| **Saturated Fat** | 0 g | 0 g |
| **Sodium** | 0.75 g | 0.28 g |
